# The First Case of Fetus in Fetu in Nicaragua: The Management Experience of the Pediatric Neurosurgery Team

**DOI:** 10.7759/cureus.33835

**Published:** 2023-01-16

**Authors:** Maria C Cantarero, Ana Lucía Osejo Cantarero, Larissa R Mendieta

**Affiliations:** 1 Pediatric Neurosurgery and Epilepsy, Hospital Infantil Nicaragua, Managua, NIC; 2 Neurosurgery, Hospital Escuela Cesar Amador Molina, Matagalpa, NIC; 3 Health Methodology, Ministry of Health of Nicaragua, Managua, NIC

**Keywords:** neural tube defect, spina bifida, meningocele, parasitic twin, fetus in fetu

## Abstract

Fetus in fetu (FIF) is a rare congenital anomaly of asymmetric monozygotic twins, where the parasitic twin develops abnormally inside the body of the host twin. In most cases, it is incorporated into the sibling’s abdomen, which frequently presents as a retroperitoneal mass. Currently, at least 200 cases have been reported worldwide, being this the first case in Nicaragua. We describe a case of a male newborn, born via cesarean section, with a history of multiple congenital malformations observed via ultrasound examination. At birth, a mass is observed on its dorsum that impresses a skull, but without the presence of bones, with three limbs, two upper and one lower, with an outline located transversely on the pelvic girdle and the presence of two male genitalia with agenesis of the testicles and an accessory kidney. A preoperative diagnosis of FIF and spinal dysraphism was made by computed tomography (CT) and magnetic resonance imaging (MRI). They shared a spinal cord and had the presence of an open spinal defect type meningocele with aberrant roots. After the diagnosis and discussion, the multidisciplinary team proceeded to surgery to perform the separation of the twin (FIF). The subsequent anatomopathological examination revealed that the fetus was anencephalic and had reliable FIF characteristics. The resection was performed followed by the closure of the 430 mL meningocele and complete separation of the spine and the parasitic twin. We present the first case of fetus in fetu in Nicaragua.

## Introduction

Fetus in fetu (FIF) [1] is a rare benign anomalous condition, defined as the presence of a parasitic, monozygotic, diamniotic fetus inside another fetus during pregnancy. In about 80% of cases, the anomalous condition occurs in the retroperitoneal region of the body of the normal fetus, although it can also occur in other areas of the body, such as the thorax, pelvis, and sacral region [2].

There is a male predominance, and most are reported before two years of age [3]. Treatment is always surgical, with the removal of the fetus with its capsule. However, the intervention of the neurosurgical team in case reports in the current literature has been limited; a major coincidence of neurosurgical involvement in this case has been the presence of meningocele, with a lack of cases describing this correlation in the scientific literature. We present the following report to emphasize the findings and treatment approach from the neurosurgical point of view and, finally, to assess the current limitations of multidisciplinary management in developing countries.

## Case presentation

**Figure 1:**
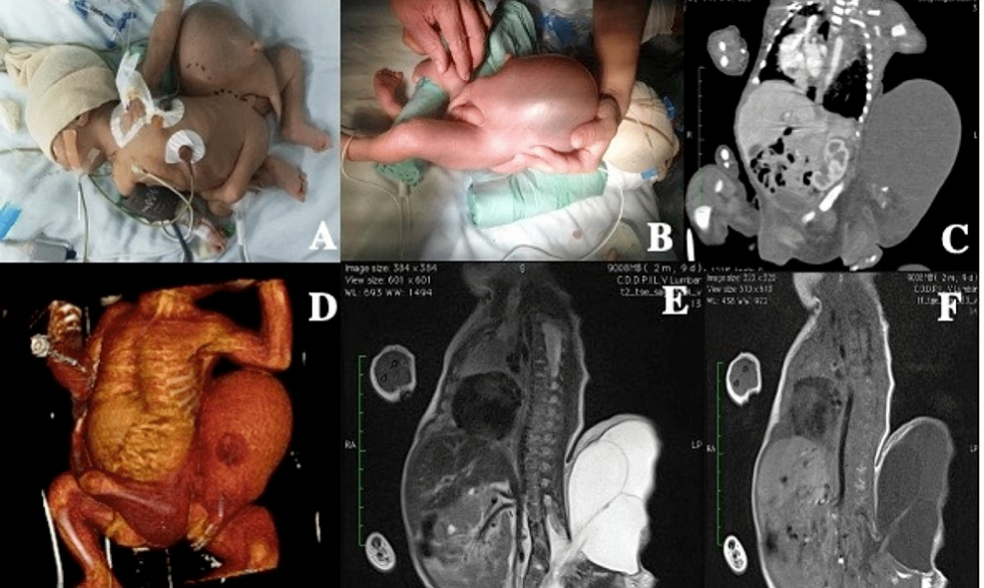
A and B: A one-week-old newborn in good general condition with paraplegia (T12-L1) with a parasitic twin attached from the dorso-lumbosacral region, amorphous, that contains rudimentary traces of the hand, right finger and left finger outline, and two extremities. C and D: Simple tomographic images and 3D reconstruction where the amorphous parasite twin with a liquid cavity and part of the extremities adhered to the newborn with the organs, viscera, muscles, and bones of the latter with adequate biology and formation. D and E: Magnetic resonance images in T1 and T2 where it is observed that both share the same column, spinal defect of the posterior elements from L1 to L2 onward, compatible with spina bifida with nerve root involvement and with a lesion compatible with myelomeningocele with multiple septal agenesis of almost the entire sacrum with a discreet vestige.

We present a male newborn of 36 + 6 weeks of gestation with a prenatal ultrasound report of multiple malformations. Physical examination showed multiple malformations (Figure 1A-1F). Ultrasound was performed and reported a pedunculated mass attached to the left lateral abdominal wall and the ipsilateral low lumbar region, which presents in its upper aspect a cystic multiloculated lesion with thin (5.9 mm) and thick (12 mm) septa and mural nodules, of which the largest measures 16 × 13 mm. The mass measures 52 × 56 × 64 mm with an approximate volume of 107 mL. In its lower aspect, there are solid areas with calcifications inside and some small cystic lesions. No vestiges of müllerian or cloacal ducts are observed.

Computed tomography (CT) angiogram of the thorax, abdomen, and pelvis reported dextrocardia, lumbar spine morphological changes at the level of the posterior elements from L2 to the most caudal portion, a sacral bone vestige, and lack of fusion of the vertebral body of L1-L2. Three iliac bones are identified, which articulate with the sacral vestige and with three femoral bones. The left lower limb is poorly formed. There is an incomplete bony pelvis where the parasitic limb articulates with dislocation of the same, which presents normal morphology.

Adhered posteriorly adjacent to the retroperitoneum is a hypodense, oval, well-defined image of a parasitic fetus, with attenuation values of 3 and -6 Hounsfield units (HU), which could correspond to a second abdominal cavity occupied by liquid fat. This image measures 97 × 83 × 93 mm, and inside, there is an image of reniform morphology measuring 35 × 24 × 30 mm, which can be related to rudimentary kidney that shows uptake of contrast medium.

On the 26th day of postnatal life, the multidisciplinary team composed of a pediatric neurosurgery, anesthesiologist, plastic surgery, orthopedics, and pediatric surgery performed a surgical intervention with the following findings: pelvic limbs, sacral remnant and atrophic hand without bone tissue, cutaneous appendix with two pedicles as a bridge that were disarticulated by orthopedics, and resections of mass of approximately 6 × 4 cm in size corresponding with supernumerary kidney, with artery, accessory renal vein, and penis with supernumerary scrotum. By pediatric surgery, it was found that it was not communicating with the healthy twin. We proceeded to perform a midline exposure from T12 to L2 through the spinal defect (bifidus), finding the absence of posterior elements, nerve roots that went to the defect sac (meningocele) containing 430 mL of yellow cerebrospinal fluid (CSF) with lumps, and dural defect of approximately 2 cm with nonfunctional roots inside; no nerve placode was found.

We proceeded to resect the meningocele, rudimentary dysfunctional roots attached to the sac, and excess dura mater. The dura was closed with 5-0 Prolene blunt sutures, thus isolating the spinal cord and spinal defect of the healthy twin. Valsalva maneuvers were performed to verify the absence of CSF fistula. The muscle and lumbosacral fascia were closed with blunt tip 4-0 Vicryl.

**Figure 2:**
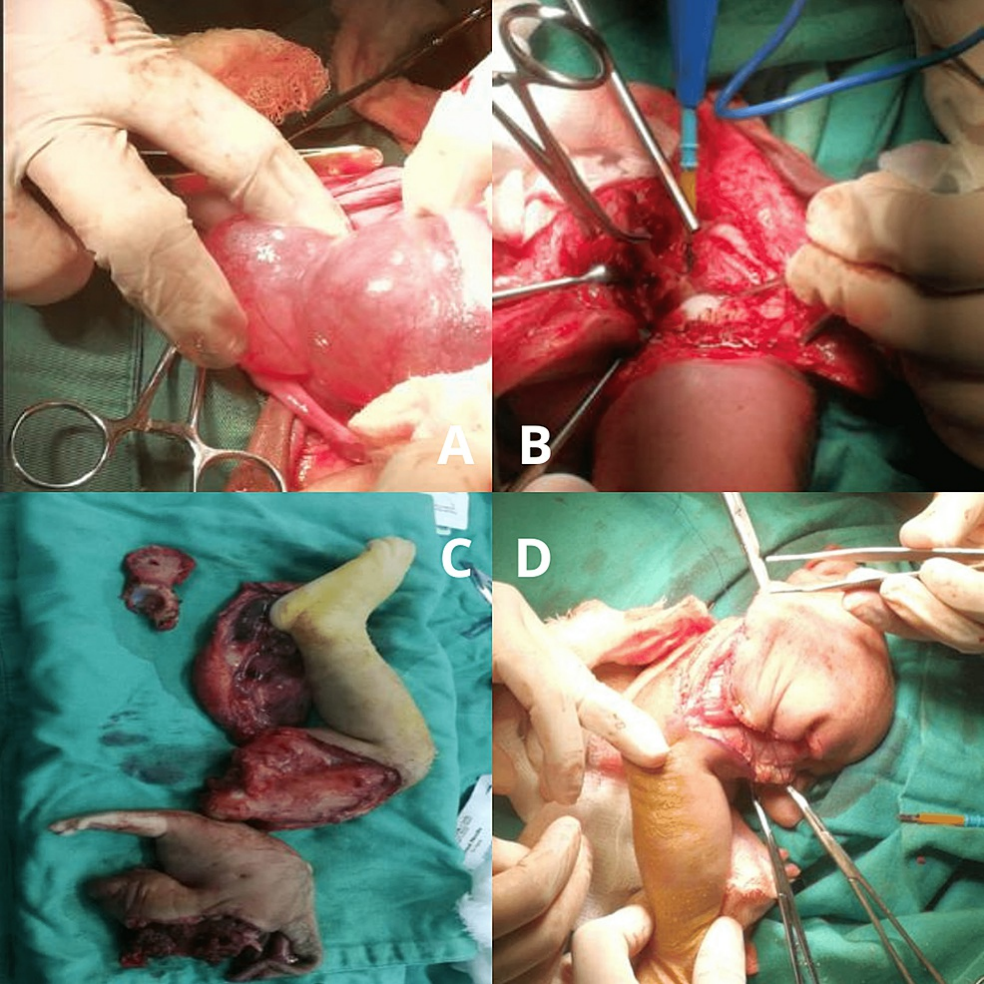
A: Exposure of the cystic cavity of the meningocele. B and C: Disarticulation and muscular vestiges of the parasitic twin. D: Myocutaneous fascia flap and closure of the entire skin defect.

Then we proceeded to resect the remaining vestiges of the skin and muscular structures of the parasitic twin (Figure 2A-2D). Plastic surgery performed a myocutaneous fascia flap and closure of the entire skin defect that represented the parasitic twin.

He was extubated from the operating room to neonatal intensive care unit, and his evolution was satisfactory. He was discharged 26 days after surgery.

## Discussion

The term “fetus in fetu” was first used by Meckel during the late 18th century as a rare condition where a malformed parasitic twin resides in the body of its host [4], with an incidence of 1 in 500,000 births [5].

FIFs are usually located retroperitoneally along the ventral midline [6], while other rare sites reported include the cerebral ventricles, liver, pelvis, scrotum [7], and mediastinum. Most often, only one fetus is present, but cases have been reported where two or more coexist [8]. This entity differs from teratomas because they are an accumulation of pluripotential cells in which there is no organogenesis or vertebral segmentation. However, the current literature has described cases where both conditions are found to coexist [9]. The distinguishing sign to differentiate a fetus in fetu from a teratoma is the presence of a separate vertebral column, which demonstrates that the fetus has passed through a primary stage after gastrulation, which involves tube formation, metamerization, and symmetrical neural development around this axis. To qualify as a FIF, one of the following features must be present: a mass enclosed within a distinct sac, partially or completely covered by skin and anatomical features recognizable to the naked eye and attached to the host by a pedicle containing a few relatively large blood vessels [10]. This case coincided with all these features, but a spinal dysraphism was identified. This was described by the finding of a lack of fusion of the vertebral body from L1 to L2 made by CT, coinciding the cystic cavity at the time of trans-surgery with an extensive meningocele of 430 mL of CSF and friable dura mater, which presents similarities with the case reported by Lu et al. [11], who described a fetus in fetu with spinal dysraphism of the host sacrum and adipose tissue in the epidural space.

Imaging tests play a key role in the diagnosis of FIF [12]. Ultrasonography is another useful aid, which allows observation of a well-delimited mass with echogenicity partially surrounded by fluids, and occasionally, a rudimentary spine is observed. CT with 3D reconstruction and MRI give additional diagnostic support [12]. Most FIF can be diagnosed during the prenatal period and before surgery. In the case reported, we had a prenatal ultrasound available, which reported multiple malformations. After birth, it was then possible to complete studies with a 3D reconstruction computed tomography in addition to MRI that confirmed the diagnosis of FIF.

Once FIF is diagnosed, surgical treatment is essential, and the prognosis is good following complete resection [13]. FIF is often enclosed in a thin fibrous capsule, covered with a single layer of epithelium or squamous epithelium. This feature of the FIF makes the tumor surrounding the tissues and organs clear, and it is easy to detach the tumor without damaging the surrounding organs and increasing complications. The success of the resection is related to a multidisciplinary surgical intervention; the participation of the pediatric neurosurgery team was fundamental for the patient’s prognosis due to their intervention in the closure of the thoracolumbosacral meningocele, closure of the cerebrospinal fluid fistula, and placement of a negative pressure drain with the objective of promoting hermetic closure of the wound, avoiding fistulas, and reducing the risk of neuroinfections [14]. In our case, sepsis of the localized cutaneous flap was presented as a complication, which was managed with surgical lavage and antibiotic therapy by plastic surgery, with satisfactory results.

Limitations

Although the various components of FIF are mature, there is still a possibility of malignant transformation. Sewell et al. reported that malignant transformation occurred after FIF resection [15]. Therefore, after surgery, close clinical observation is necessary. The patient should be carefully monitored, and alpha-fetoprotein (AFP) or β-human chorionic gonadotropin (β-HCG) levels should be evaluated in combination with imaging to detect any early signs of malignancy [15]. In our case, this was a limitation since we did not have the availability of such biomarkers, and the follow-up was only clinical.

## Conclusions

Although FIF is a congenital malformation that is rarely encountered in clinical practice, it is potentially harmful to the human body, especially when the patient is a newborn. The development of prenatal diagnostic techniques and the popularization of imaging technology have increased the rate of diagnosis of FIF. The multidisciplinary approach and intervention of the pediatric neurosurgery specialty were of great importance since intervening on the associated neural tube defect decreased neuroinfectious complications, improving neurodevelopment and the quality of life of the child and family.
